# A Tale of Two Cysts: A Histopathological Case Report of Epithelial and Mesothelial Splenic Cysts

**DOI:** 10.7759/cureus.75200

**Published:** 2024-12-06

**Authors:** George S Stoyanov, Andreya Kirilova, Kristina Naydenova, Zlatko Zlatev, Hristo Popov, Radoslav Georgiev, Kiril Kirov

**Affiliations:** 1 Pathology, Multiprofile Hospital for Active Treatment, Shumen, BGR; 2 Cytopathology, Diagnostic and Consultative Center, Shumen, BGR; 3 General and Clinical Pathology, Forensic Medicine and Deontology, Medical University of Varna, Varna, BGR; 4 Radiology, St. Marina University Hospital, Varna, BGR; 5 Research Institute, Medical University of Pleven, Pleven, BGR

**Keywords:** cyst, epithelial cyst, mesothelial cyst, spleen, tumor

## Abstract

Splenic cysts are rare medical conditions, and their incidence is dominated by parasitic types. Non-parasitic splenic cysts, whether true cysts (with a cellular lining of the cystic wall) or pseudocysts (without a cellular lining), are significantly rarer than parasitic ones. Their etiology is not fully established, with fetal remnant development, metaplasia, and mesothelial invagination being widely accepted possible mechanisms. Splenic cysts are rarely symptomatic if small and are predominantly discovered incidentally, while larger and multiple splenic cysts mainly present with dull abdominal pain or discomfort. Herein, we report two cases of splenic cysts. The first case involves a 14-year-old female with an insignificant medical history, presenting with dull abdominal pain developing over the previous month. Computed tomography (CT) revealed a cystic lesion within the lower aspect of the spleen, measuring 150 × 130 × 115 mm, with compression of the left kidney. The patient was treated with partial splenectomy, and histopathology revealed a true epithelial cyst. The second case involves a 45-year-old male, also without significant prior medical history, presenting with subacute abdominal pain. Abdominal CT showed multiple splenic cysts, the largest measuring 50 mm, and multiple dispersed smaller ones measuring between 4 and 8 mm, with compression of the left kidney. The patient was treated with total splenectomy, and histopathology showed multiple mesothelial splenic cysts.

## Introduction

Splenic cysts are a broad group of conditions with varying etiopathogenesis. Altogether, their incidence is low and dominated by parasitic ones, with an overall incidence of 0.07% in the general population and only around 1,000 cases reported so far for non-parasitic ones [1-5]. Depending on their etiopathogenesis, splenic cysts are subdivided into parasitic and non-parasitic types. Parasitic cysts are dominated by echinococcosis (hydatid cysts) caused by *Echinococcus granulosus* and, less often, by *Echinococcus multilocularis* (fox tapeworm) [6].

Non-parasitic splenic cysts, while being significantly less common, as already mentioned, have a broader etiopathogenesis and are classified into true cysts and pseudocysts [1,7]. Separation into true or pseudocyst is based on the presence of a cellular lining of the cystic wall [1,7,8]. Pseudocysts are reportedly more common than true cysts, with their etiology often linked to incomplete repair following splenic injuries such as trauma with hematoma formation, infarction, or infection leading to abscess cavity formation [1,2,9]. In such cases, the cystic cavity is often filled with debris, inflammatory cells, or blood components, and the pseudocyst wall consists of dense connective tissue, often with calcification [9].

True splenic cysts represent less than 10% of non-parasitic ones and are most often diagnosed in children or young adults, though cases in older individuals have also been reported [1,2,9]. These cysts represent a mixed group of conditions, ranging from congenital anomalies to tumors. Cystic tumors of the spleen include cystically transformed hemangiomas and lymphangiomas, splenic cystadenomas and cystadenocarcinomas, as well as cystic metastases, predominantly from gastrointestinal tract adenocarcinomas [2,9,10]. Non-tumorous cysts include true epithelial and mesothelial cysts, which are congenital in nature and are thought to develop either from mesothelial invagination and metaplasia or from endodermal remnants within the spleen [5]. Herein, we report two cases of true splenic cysts: one epithelial and one mesothelial.

## Case presentation

Case 1

A previously healthy 14-year-old female presented with complaints of abdominal discomfort that had gradually developed over the previous month. Outpatient laboratory tests were insignificant in findings, and abdominal ultrasound and CT revealed a cystic lesion within the lower aspect of the spleen with compression of the left kidney. The cyst measured 150 x 130 x 115 mm. Despite the insignificant result of peripheral blood, under suspicion of echinococcosis, surgical intervention was scheduled. Two accessory spleens, each measuring 1 cm in diameter, were noted in the splenic hilum during the procedure. The cystic lesion was aspirated, and the content showed a viscous brownish fluid. As the aspiration finding did not comply with echinococcosis, an open partial splenectomy was undertaken. The postoperative period was uneventful, with full symptom resolution.

**Figure 1:**
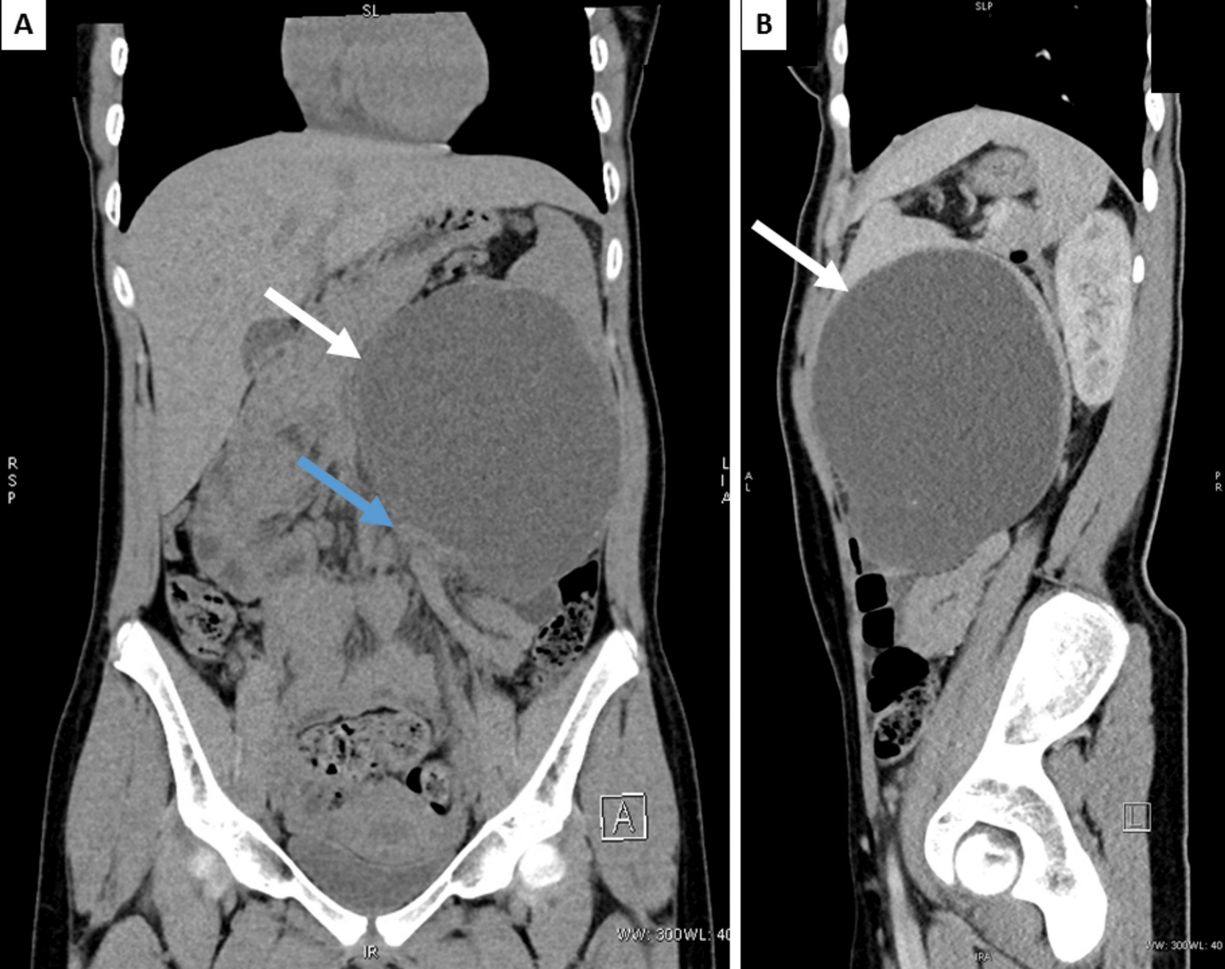
Abdominal computed tomography image A: Coronal view with a cystic lesion of the spleen (arrow) and renal compression (blue arrow); B: sagittal view with a smooth-walled cyst in the lower part of the spleen (arrow).

The resected specimen weighed 102 g and had a grossly preserved splenic parenchyma rim. The cystic wall was thick and grayish, with trabeculations.

**Figure 2:**
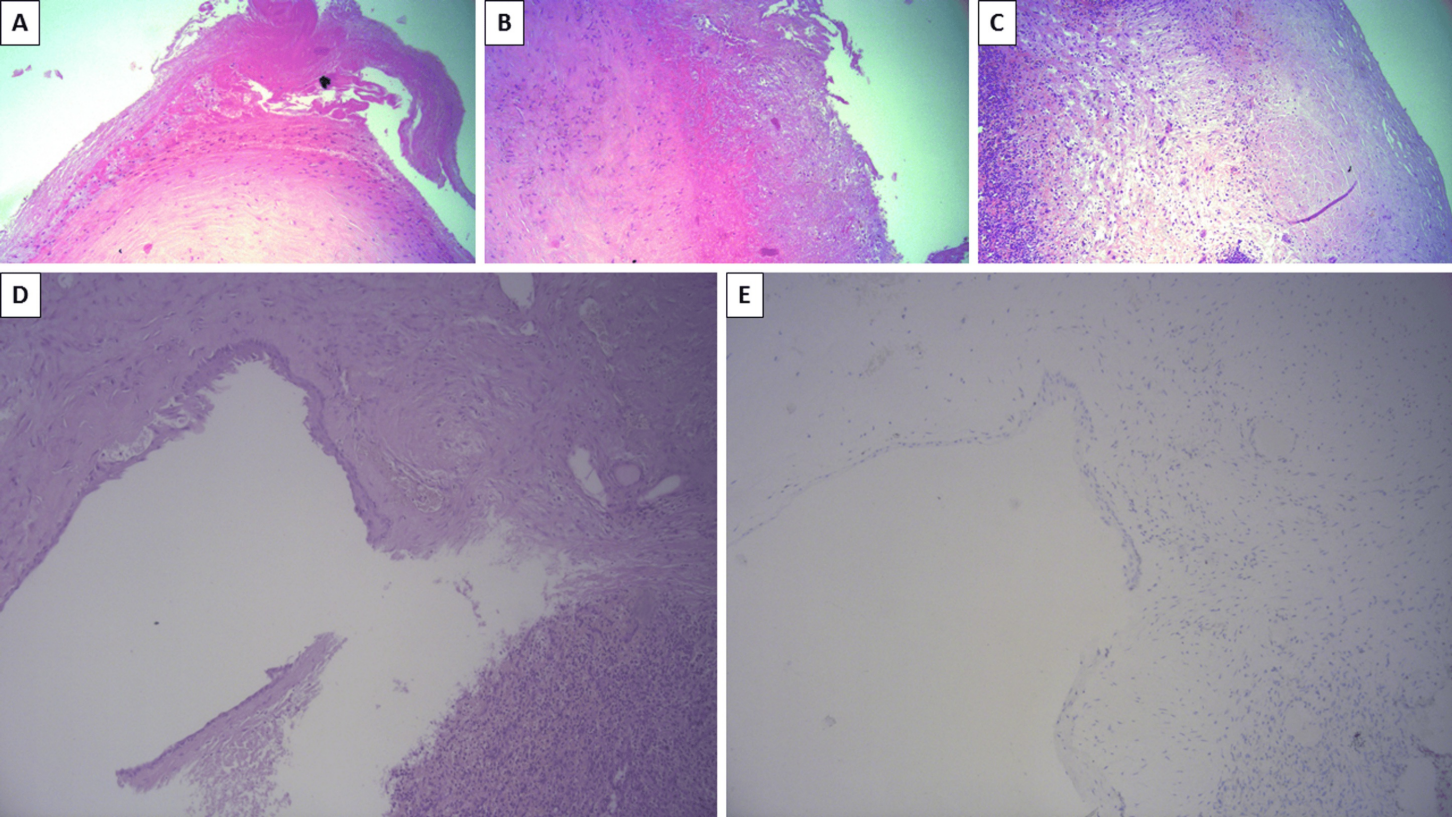
Splenic cyst morphology and immunohistochemistry A and B: A thick fibrous wall with epithelial denudation and fibrin deposits; C: the fibrous wall of the cyst and neighboring splenic parenchyma with reactive changes; D: the stratified epithelial lining of the cyst; E: negative immunohistochemical reaction for calretinin in the epithelial lining. A-D: hematoxylin and eosin stain; original magnifications x100.

Histology of the specimens showed a splenic cyst with a thick fibrous wall, covered by multilayered flat epithelium akin to stratified squamous, areas of accommodate monolayer epithelium and areas of complete epithelial denudation, possibly from the intraoperative cyst aspiration (Figure 2 A-D). Calretinin immunohistochemistry was negative in the epithelial lining of the cyst; hence, the diagnosis of a true epithelial splenic cyst was established as mesothelial differentiation was disproved (Figure 2E).

Case 2

A previously healthy 45-year-old male presented with subacute abdominal pain. Outpatient consultation with gastroenterology showed palpatory pain in the left upper abdominal quadrant and a positive stool test for *Helicobacter pylori*. Abdominal ultrasound and CT showed multiple splenic cysts, with the largest measuring 50 mm and several smaller ones ranging from 4 to 8 mm, along with compression of the left kidney. Due to the multitude of cysts and under suspicion for echinococcosis, an open total splenectomy was scheduled, which went uncomplicated and the postoperative period was uneventful, with full symptom resolution.

**Figure 3:**
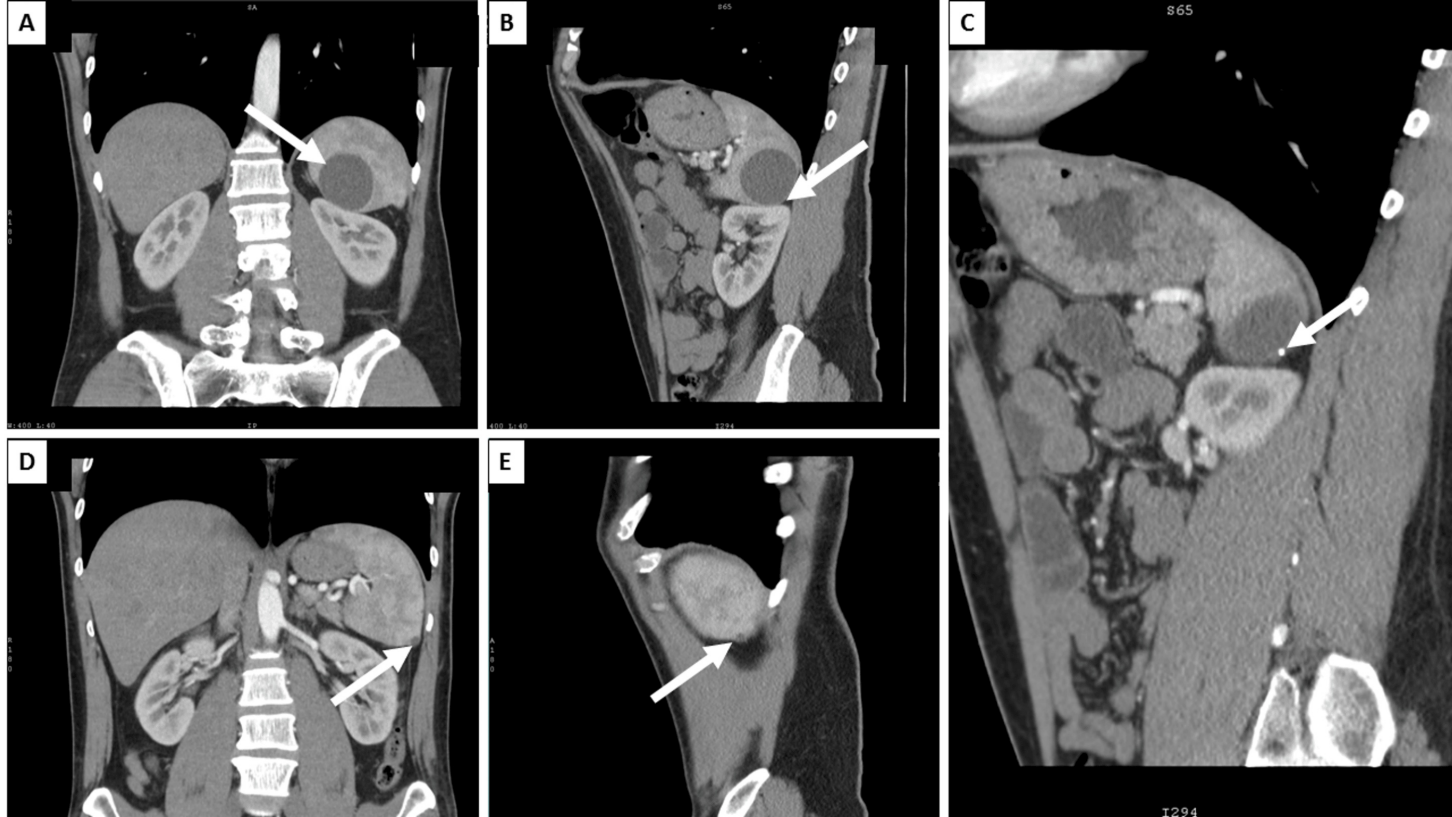
Contrast-enhanced computer tomography A: Coronal view with a non-enhancing cystic lesion of the spleen (arrow); B: sagittal view with kidney compression (arrow); C: sagittal view with calcifications within the wall of the cyst (arrow); D: coronal view with smaller subcapsular cyst (arrow), axial view; E: sagittal view of the smaller subcapsular cyst (arrow)

**Figure 4:**
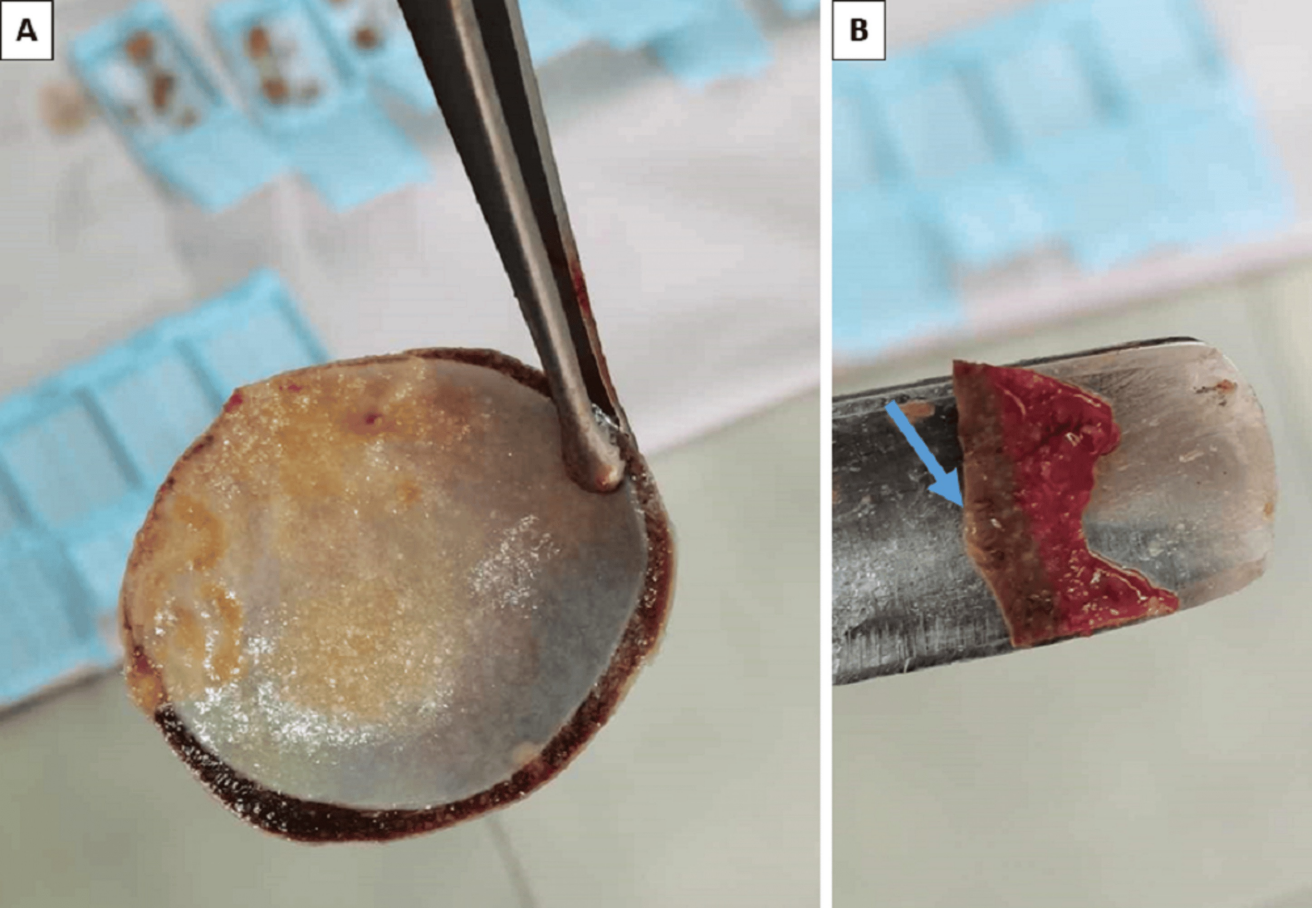
Splenic cyst A: Fibrous wall of the large cyst with a smooth inner cyst lining and yellowish fluid within the cyst; B: smaller subcapsular cyst (arrow) with a thin wall and serous content.

The resected spleen measured 15 × 10 × 6 cm, weighed 295 g, and exhibited multiple subcapsular cysts with a gelatinous appearance, ranging in size from 2 to 8 mm. On sectioning, a cyst located at the lower pole measured 6 cm in diameter, with a thick, smooth fibrous wall and filled with yellowish fluid (Figure 4).

**Figure 5:**
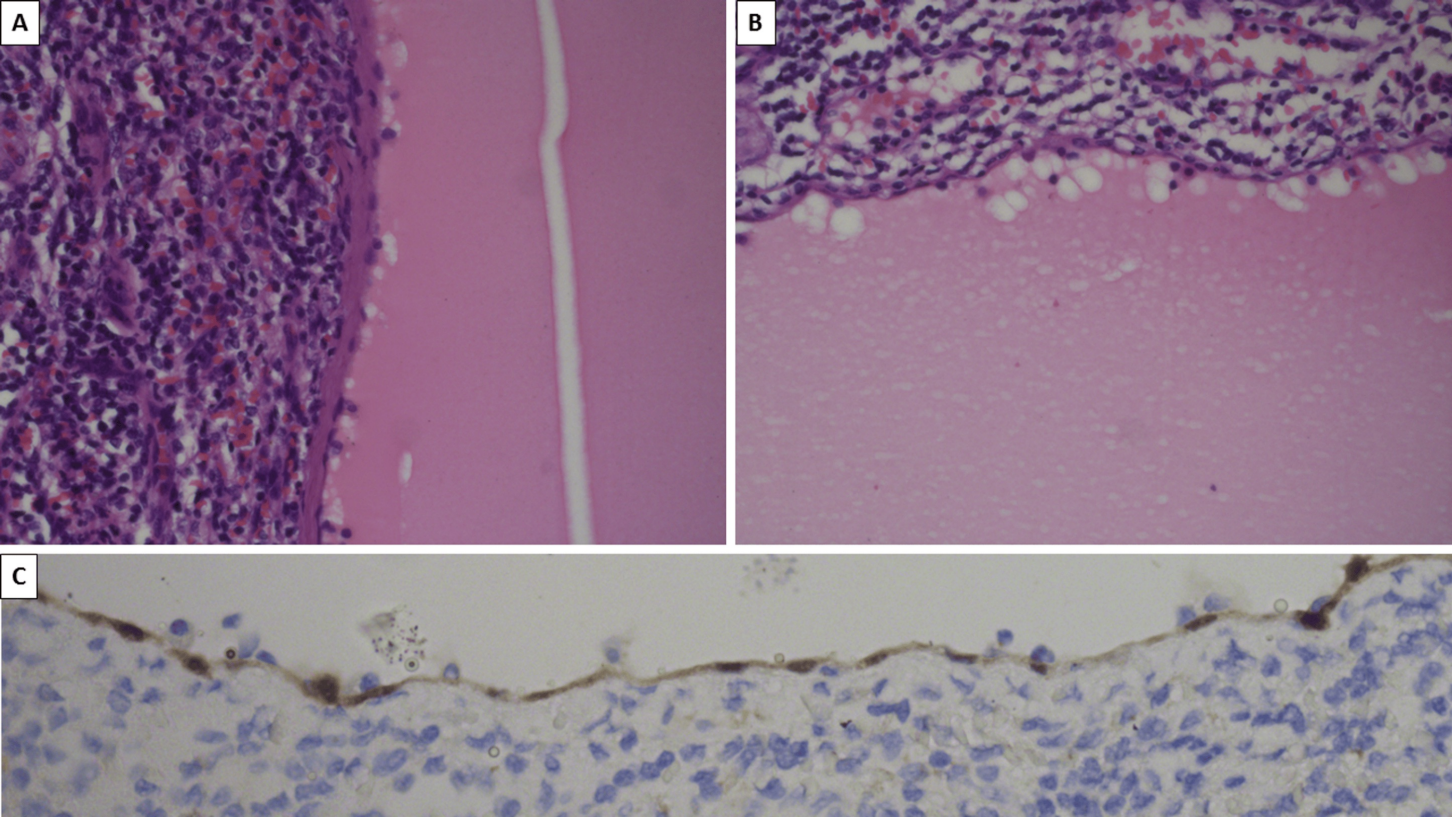
Splenic cyst morphology and immunohistochemistry A: Fibrous wall covered with a monolayer of flattened cells; B: reactive changes in the neighboring splenic parenchyma and resorption artifact of the cystic wall lining; C: positive immunohistochemical reaction for calretinin of the cystic wall lining. A and B: hematoxylin and eosin stain; original magnifications x400.

Histopathological examination of the specimen revealed splenic parenchyma with a thick fibrous-walled cyst (wall thickness up to 5 mm) showing hyaline degeneration and focal calcifications. The cyst was lined by a flattened, monolayered, and monomorphic cellular lining, likely mesothelial in origin. Multiple small subcapsular cysts were also noted, characterized by a thin fibrous wall and an identical lining to that of the large cyst. The cavities of these subcapsular cysts were filled with a colloid-like fluid, occasionally admixed with foamy macrophages and resorption vacuoles (Figure 5A,B). Immunohistochemistry for calretinin was positive in the cyst lining, confirming the diagnosis of multiple mesothelial splenic cysts (Figure 5C).

## Discussion

Splenic cysts, both true and pseudocysts (non-parasitic ones), are rare conditions with a not fully established etiology [2]. While some entries, such as cystic hemangioma and lymphangioma, are true benign neoplastic processes and cystic metastases are metastatic malignant tumors, some other entries remain more dubious in their origin [5]. Mesothelial cysts are thought to arise either from fetal remnant tissue or from mesothelial invagination [5,9,10]. Both of these theories are plausible as most of the mesothelial cysts are multiple, as seen in the second of our presented cases, and are predominantly located in the subcapsular region. Furthermore, in our first case, the patient had one of the most common congenital abdominal aberrations: accessory spleens (spleniculi) [11]. Prior abdominal trauma or surgery can also play a role in mesothelial cyst development, but most patients with such cysts report either no history of abdominal trauma or only minor and insignificant ones [7,10]. Epithelial cysts are thought to possibly originate from mesothelial ones with epithelial metaplasia of the cystic lining or as direct evolution of fetal remnants akin to true dermoid/epidermoid cysts [5,9,10].

The true incidence is difficult to establish as small cysts are asymptomatic and can only be discovered as an incidental finding in medical evaluations for another cause: ultrasound, CT, or laparoscopy [2,5,7,9]. Symptoms arise in larger cysts, generally larger than 5 cm, and are predominated by blunt pain and abdominal discomfort due to the mass effect on neighboring structures [2,7,9,12,13].

For incidental, non-symptomatic, and small cysts, the general management of the condition is that of regular follow-up, as the risks of the surgical procedure far outweigh the potential benefits [1,2,4,5,7,8,10,12,13]. If symptomatic or larger, surgical intervention should be considered, with partial splenectomy preferred for singular and small-to-medium-sized cysts and total splenectomy for larger and especially multiple cysts or those located in the splenic hilum [1,2,5,7,10,13].

Recovery after surgery is usually favorable in cases of non-parasitic and non-metastatic cysts, with the risk of complication being relative to other abdominal surgeries in non-malignant and non-infectious conditions [1,5,12,13].

## Conclusions

Non-parasitic splenic cysts have varying and widely unestablished etiologies. As seen in the presented cases, true splenic cysts can produce largely non-specific symptoms based predominantly on their size and displacement of neighboring organs. In contrast, smaller cysts are generally asymptomatic and are often discovered as incidental findings. For symptomatic and especially large cysts, surgical treatment by either partial or complete splenectomy is the treatment of choice. Definitive subtyping of splenic cysts is carried out on histology and is dependent on the presence or lack thereof of cells covering the cystic wall, their morphology, and immunophenotype. As such, non-parasitic splenic cysts have a complex and diverging classification into either tumors or developmental anomalies. A lack of World Health Organization classification further underlines the rarity of these conditions.
